# METTL3 regulated by histone lactylation promotes ossification of the ligamentum flavum by enhancing the m6A methylation of BMP2

**DOI:** 10.1186/s10020-025-01173-x

**Published:** 2025-03-25

**Authors:** Jiaming Zhou, Rui Wang, Zequn Zhang, Yuan Xue

**Affiliations:** 1 Department of Orthopaedic Surgery, Tianjin Key Laboratory of Spine and Spinal Cord, Tianjin Medical University General Hospital, Tianjin, China; 2 Tianjin Key Laboratory of Spine and Spinal Cord, Tianjin Medical University General Hospital, Tianjin, China

**Keywords:** Ossification of the ligamentum flavum, m^6^A methylation, Histone lactylation, METTL3, BMP2

## Abstract

Ossification of the ligamentum flavum (OLF) is characterized by ligamentum flavum thickening and subsequent thoracic canal stenosis. Emerging evidence has demonstrated the involvement of N6-methyladenosine (m6A) methylation in OLF pathogenesis. This study investigates the regulatory role of METTL3-mediated m6A methylation of BMP2 in OLF progression. Clinical ligamentum flavum tissues were analyzed for m6A levels using dot blot analysis. Osteogenic differentiation was assessed through quantitative real-time PCR (qPCR), alkaline phosphatase staining, alizarin red S staining, and western blot analysis. Mechanistic insights were obtained through methylated RNA immunoprecipitation (MeRIP), RNA immunoprecipitation (RIP), and luciferase reporter assays. The regulatory role of histone lactylation on METTL3 expression was examined using LDHA knockdown, sodium lactate (Nala) treatment, and 2-deoxy-D-glucose (2-DG) administration in OLF cells. Our findings revealed significant upregulation of METTL3 expression and m6A levels in OLF patients. METTL3 was shown to enhance osteogenic differentiation and m6A methylation of BMP2, which was specifically recognized by IGF2BP1. Furthermore, increased histone lactylation was observed in OLF patients, with enrichment in the METTL3 promoter region facilitating its transcriptional activation. LDHA knockdown-mediated inhibition of endogenous lactylation suppressed osteogenic differentiation, a phenotype that was rescued by METTL3 overexpression. In conclusion, this study elucidates that histone lactylation-mediated upregulation of METTL3 promotes OLF progression through IGF2BP1-dependent m6A methylation of BMP2, providing novel insights into potential therapeutic strategies for OLF management.

## Introduction

Ossification of the ligamentum flavum (OLF) can lead to a narrowing of the thoracic canal, resulting in a series of symptoms, including spinal cord and nerve compression, as well as numbness in the lower extremities (Ren et al. 2013). Although several studies suggest that genetic, metabolic, and growth factors may contribute to the progression of OLF, the pathogenesis of OLF remains unclear (Takahashi et al. 2018; Uchida et al. 2011). Currently, there is no effective method to prevent the progression of OLF, and surgery remains the primary treatment option (Machino et al. 2022). Therefore, it is crucial to further explore the pathogenesis of OLF and identify potential therapeutic targets for its prevention and treatment.

N6-methyladenosine (m^6^A) RNA methylation is one of the most common RNA modifications, regulating multiple biological functions, including embryonic stem cell maintenance and differentiation, transcriptional splicing, protein translation control, heat shock response, meiosis, and neuronal function (An and Duan 2022). m^6^A modification is tightly regulated by methyltransferases (writers), demethylases (erasers) and binding proteins (readers) (Zaccara et al. 2019). Notably, m^6^A modification is catalyzed by a methyltransferase complex comprising METTL3, METTL14, and WTAP. METTL3, one of the most important components of the m^6^A methyltransferase complex, is primarily responsible for catalyzing the m^6^A modification of RNA molecules (Du et al. 2018). Several studies have reported the role of METTL3-mediated m6A methylation in osteogenic differentiation (Tian et al. 2019; Yu et al. 2020; Song et al. 2021). In OLF, m^6^A methylation mediated by ALKBH5 has been shown to regulate its development (Wang et al. 2020). However, the function of METTL3 in the development of OLF remains unclear.

Bone morphogenetic proteins (BMPs) are the main factors that induce bone and cartilage formation in vivo (Lowery and Rosen 2018). BMP-mediated Smad signaling plays a central role in regulating many transcription factors, including Runx2, Osterix and MSX2, which are essential for osteoblast formation and chondrogenesis (Yahiro et al. 2020; Yan et al. 2022; Liu et al. 2020). Previous studies have revealed that BMP2 m6A modification mediates osteogenic differentiation (Wang et al. 2020; Liu et al. 2021). In addition, the expression of BMP2 is upregulated in patients and rats with OLF, indicating an association between BMP2 and the development of OLF (Qu et al. 2017; Zhao et al. 2021). However, how BMP2 m6A methylation mediates the progression of OLF remains to be further investigated.

Protein post-translational modifications (PTMs) covalently modify amino acid residues by adding modification groups or removing them through protein hydrolysis (Macek et al. 2019). Histone lactylation is a recently discovered PTM mediated by lactate, linking cellular metabolism with gene regulation and playing an important role in various diseases (Li et al. 2022). In malignancy, lactate is an important metabolite of glycolysis that regulates the lactylation modification of downstream proteins (Wang et al. 2023). Lactylation of histone 3 on lysine residue 18 (H3K18la) is highly enriched at gene promoters and correlates with active gene expression of associated genes in macrophages (Zhang et al. 2019). In recent years, one study demonstrated that lactylation-driven METTL3-mediated RNA m6A modification promotes immunosuppression in tumor-infiltrating myeloid cells (Xiong et al. 2022). Moreover, histone lactylation in bone mesenchymal stem cells has been shown to promote osteogenic differentiation (Wu et al. 2023). However, whether lactylation is involved in the development of OLF remains uncertain.

In this study, we investigated the influence of histone lactylation-regulated METTL3 on BMP2 m^6^A methylation and its impact on the development of OLF. These results provide a new theoretical basis for the treatment of OLF.

## Methods

### Ethical statement

The study was carried out in accordance with the principles of the Declaration of Helsinki. Approval was granted by the Ethics Committee of Tianjin Medical University General Hospital. All individuals involved in the study received informed consent. All methods were carried out in accordance with relevant guidelines and regulations.

### Sample collection

The ligamentum flavum tissues were obtained from 20 patients with OLF who underwent spinal decompression surgery for myeloradiculopathy, and 18 non-OLF patients who had surgery for thoracic spine trauma caused by external force. The inclusion criteria for patients with OLF are as follows: (1) symptoms of spinal cord compression such as limb numbness, reduced sensation, and lower limb weakness; (2) X-ray and MRI examinations show ossification of the ligamentum flavum in the posterior spinal canal; (3) intraoperative confirmation of thoracic ligamentum flavum ossification, with no contamination of the ligamentum flavum tissue. The inclusion criteria for non-OLF patients are as follows: (1) patients admitted during the same time period with a definite diagnosis of thoracic vertebral fracture; (2) no ossification of ligamentum flavum was found in clinical symptoms, imaging findings and intraoperative findings. Participants signed informed consent, and this study was approved by the hospital.

### Cell culture and treatment

OLF cells were isolated from the ligamentum flavum tissues obtained from patients with OLF. Cell isolation and culture were performed according to a previous report (Specchia et al. 2001). Briefly, tissues were placed in phosphate-buffered saline (PBS; Gibco, Grand Island, NY, USA) and cut into pieces of approximately 0.5 mm^3^ under aseptic conditions. The collagenase-treated pieces were cultured in DMEM/F-12 medium (Gibco) containing 10% fetal bovine serum (FBS, Gibco), 100 U/mL penicillin and 100 µg/mL streptomycin in a 5% CO_2_ incubator at 37℃ for 2–3 weeks, until cells migrated from ligaments to become a monolayer. Third passage OLF cells were used for experiments.

To investigate the effect of histone lactylation on METTL3, cells were treated with 5 mM histone lactylation inducer sodium lactate (Nala; Merck, Darmstadt, Germany) or 10 mM histone lactylation inhibitors 2-deoxy-D-glucose (2-DG; Merck) for 24 h for the subsequent experiments.

To induce osteogenic differentiation of OLF cells, cells were cultured in DMEM/F-12 containing 10% FBS, 25 µg/ml ascorbic acid, 10 mM β-glycerol phosphate and 10 nM dexamethasone for 2 weeks.

### Cell transfection

Cells were seeded in the six-well plate when cell fusion reached 60–80%. Next, cells were transfected with short hairpin RNA targeting (sh) METTL3, YTHDF1, YTHDF2, YTHDF3, YTHDC1, YTHDC2, IGF2BP1, IGF2BP2, IGF2BP3 and LDHA, shRNA negative control (shNC), BMP2 overexpressing plasmids, METTL3 overexpressing plasmids and empty vector (pcDNA3.1) (All provided from GenePharma, Shanghai, China) using Lipofectamine 2000 (Invitrogen, Carlsbad, CA, USA) according to the guidelines. After 48 h of transfection, the transfected cells were harvested.

### Dot blot assay

Total RNA extraction from ligamentum flavum tissues was conducted using Trizol reagent (Invitrogen). RNA was heated on a PCR instrument at 95℃ for 5 min for denaturation, loaded on a Hybond-N + membrane and UV cross-linked at 302 nm. Subsequently, the membrane was blocked with 5% skim milk for 2 h and incubated with anti-m6A overnight at 4 ℃ and secondary antibodies for 2 h at room temperature, respectively. The membrane was visualized using an enhanced chemiluminescence (ECL) kit (Thermo Scientific). For internal standard detection, the membrane was stained with 0.02% methylene blue (Sigma-Aldrich, St. Louis, MO, USA) for 10 min for observation.

### Quantitative real-time PCR (qPCR)

Trizol reagent (Invitrogen) was used to isolate total RNA from the ligamentum flavum tissues and OLF cells. Next, cDNAs were synthesized using a 1st Strand cDNA Synthesis Kit (Takara, Shiga, Japan). qPCR was performed on the obtained cDNA samples using SYBR green master mix (Thermo Scientific, Waltham, MA, USA). Relative mRNA expression was calculated using the 2^−ΔΔCt^ method as normalized to GAPDH.

### Alkaline phosphatase (ALP) staining

The ALP staining intensity in OLF cells was assessed using a BCIP/NBT alkaline phosphatase color development kit (Beyotime, Beijing, China). Cells were stained with BCIP/NBT staining solution for 30 min protected from light at room temperature. After staining, cells were washed with distilled water and photographed.

### Alizarin red S (ARS) staining

The calcium deposition in OLF cells was evaluated using an alizarin red S staining kit (Beyotime). Cells were fixed in the fixing solution for 20 min and then stained with ARS solution for 30 min. After staining, cells were washed with distilled water and photographed.

### Western blot assay

Total protein was isolated using radio immunoprecipitation assay (RIPA) lysis buffer (Thermo Scientific) and measured by a BCA kit (Beyotime). Protein samples were loaded on 10% SDS-PAGE for electrophoresis, and then transferred to PVDF membranes. The membranes were blocked with 5% skimmed milk for 1 h, and incubated overnight at 4℃ with anti-OPN (1: 1000, ab214050, Abcam, Cambridge, UK), anti-OCN (1: 1000, ab133612, Abcam), anti-Runx2 (1: 1000, ab236639, Abcam), anti-Smad1/5/8 (1: 1000, PA5-104523, Thermo Scientific), anti-p-Smad1/5/8 (1: 1000, PA5-64711, Thermo Scientific), anti-GAPDH (1: 10000, ab181602, Abcam), and anti-H3K18la (1: 1000, PTM-1427RM), anti-kla (1: 1000, PTM-1401RM, PTM BIO, Hangzhou, China) and anti-histone H3 (1: 1000, PTM-841RM, PTM BIO). Afterwards, membranes were incubated with secondary antibodies (1: 10000, ab6721, Abcam) for 2 h. Blots were visualized using the ECL reagent (Yeasen, Shanghai, China) and photographed with an optical luminescence instrument.

### Methylated RNA Immunoprecipitation (MeRIP)

The m^6^A levels of BMP2 were assessed using a Magna MeRIP m^6^A kit (Merck). In brief, the anti-m^6^A antibody was pre-incubated with 50 µl beads in IP buffer for 1 h at room temperature. Next, fragment RNAs were added to the antibody-beads mixture and incubated at 4 °C for 4 h on a rotator. Then, the mixture was washed with IP buffer and digested using proteinase K, and the bound RNAs were extracted using phenol-chloroform method. The expression of BMP2 was measured by qPCR.

### RNA Immunoprecipitation (RIP)

The RIP assay was conducted using an imprint RNA immunoprecipitation kit (Sigma-Aldrich, St. Louis, MO, USA). To evaluate the interaction between METTL3 and BMP2, OLF cells were lysed in RIP lysis buffer, and the lysate was incubated overnight at 4℃ with the anti-METTL3 or anti-IgG coated magnetic protein A/G beads. After purification, the expression of BMP2 was detected by qPCR.

### RNA-sequencing (RNA-seq) and bioinformatic analysis

Total RNA of OLF cells transfected with shNC or shMETTL3 was extracted and for RNA-seq by Novogene (Beijing, China). The differentially expressed genes (DEGs) were defined as *P* < 0.05 and|log (fold change)| > 2. The potential m^6^A methylation sites of BMP2 were predicted using the SRAMP database (http://www.cuilab.cn/sramp).

### Dual luciferase reporter assay

To detect which sites are the m^6^A methylation sites of BMP2, wild type (WT)-BMP2 and mutant (MUT)-BMP2 sequences containing METTL3 methylation sites were cloned into the Pmir-GLO vector to construct luciferase reporter plasmids. OLF cells were seeded in 96-well plates and co-transfected with the plasmids with shNC, shMETTL3, shIGF2BP1, shLDHA, pcDNA3.1 or pcDNA3.1-METTL3 using Lipofectamine 2000 reagent for 24 h. After transfection, dual luciferase reporter assay system was used to evaluate the luciferase activity (Promega, San Luis Obispo, CA, USA). The intensity of the firefly luciferase was normalized to Renilla luciferase signal intensity.

### RNA stability detection

OLF cells after transfection were treated with 5 µg/mL actinomycin D (Merck) for 1, 4, 8 and 12 h, and the expression of BMP2 mRNA was measured by qPCR to assess the stability of BMP2.

### RNA pull down

RNA pull down was performed using a RNA pull down assay kit (Sangon, Shanghai, China). Cells were harvested and lysed in IP lysis buffer containing protease inhibitor, followed by sonication and centrifugation to obtain the supernatant. Streptavidin magnetic beads were washed and incubated with biotinylated RNA probes for 1–2 h at room temperature. The RNA-bead complexes were then mixed with cell lysate, binding buffer, and glycerol, and incubated overnight at 4 °C. After incubation, the beads were washed with Wash Buffer I and Wash Buffer II. For protein analysis, the complexes were eluted by boiling in Elution Buffer, and the supernatant was collected for downstream applications. For RNA analysis, Trizol was added to extract RNA, which was precipitated with isopropanol, washed with ethanol, and dissolved in DEPC water.

### Chromatin Immunoprecipitation (ChIP)

A ChIP assay kit (Beyotime) was performed to evaluate the H3K18la enrichment on METTL3 promoter. Briefly, cells were fixed with 1% formaldehyde for 10 min and then sonicated. Afterwards, cells were centrifuged at 4℃ and 12,000×g for 5 min to get supernatant. Next, the supernatant was mixed with 70 µl Protein A + G Agarose/Salmon Sperm DNA for 30 min at 4℃. Then, the mixture was centrifuged at 10,000×g for 1 min, and the obtained supernatant was incubated with 1 µg anti-H3K18la overnight at 4℃. Next, Protein A + G Agarose/Salmon Sperm DNA (60 µl) was added to the mixture and rotated for 60 min at 4℃. Afterwards, the mixture was centrifuged for 1 min at 1000×g and 4℃. After removing the supernatant, the precipitate was washed with buffer and detected by qPCR.

### Statistical analysis

GraphPad Prism 7 software was used to analyze and process the data. Results were expressed as mean ± standard deviation of at least three replicates. The comparison between two or more groups was analyzed using student’s t-test or one-way analysis of variance (ANOVA). *P* < 0.05 was recognized as statistically significant.

## Results

### METTL3 expression and m^6^A level are upregulated in patients with OLF

**Fig. 1:**
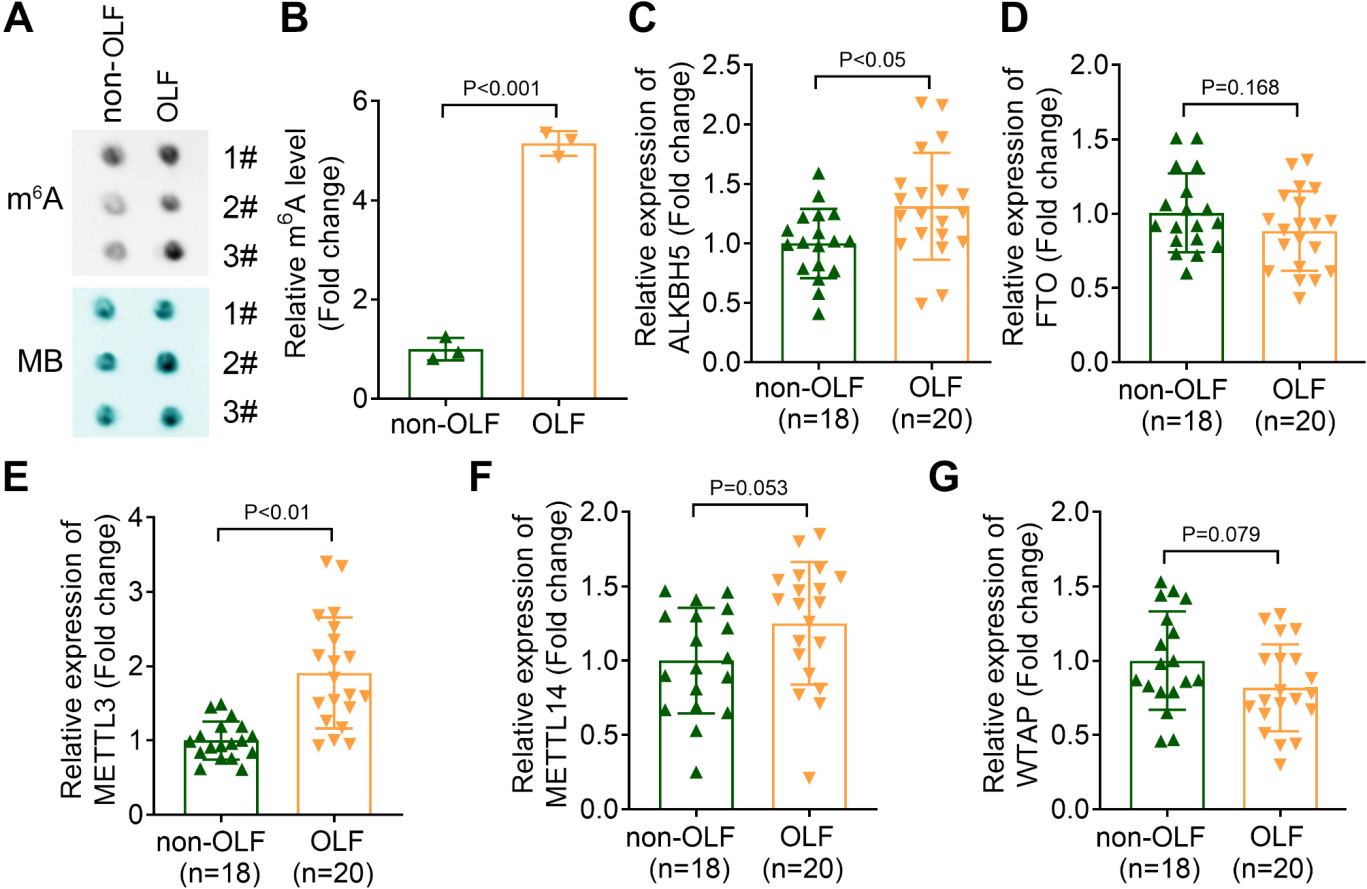
METTL3 expression and m^6^A level were upregulated in patients with OLF. (**A** and **B**) The m^6^A levels in ligamentum flavum tissues in patients with or without OLF were detected by dot blot assay. The expression of demethylases (**C**) ALKHB5, (**D**) FTO and methyltransferases (**E**) METTL3, (**F**) METTL14 and (**G**) WTAP in patients with OLF and non-OLF was measured by qPCR

To elucidate the mechanism by which m6A modification regulates OLF development, we assessed the levels of m6A methylation and the expression of demethylases ALKBH5 and FTO, as well as methyltransferases METTL3, METTL14, and WTAP, in patients with OLF compared to those without OLF. Our results demonstrated that m6A methylation was significantly increased in OLF patients (Fig. 1A and B). Furthermore, the expression levels of ALKBH5 and METTL3 were markedly elevated in OLF patients, with METTL3 showing the most pronounced increase. However, no significant differences were observed in the expression levels of FTO, METTL14, and WTAP between the two groups (Fig. 1C-G). Consequently, METTL3 was selected for further investigation in subsequent experiments.

### METTL3 knockdown inhibits osteogenic differentiation in OLF cells

**Fig. 2:**
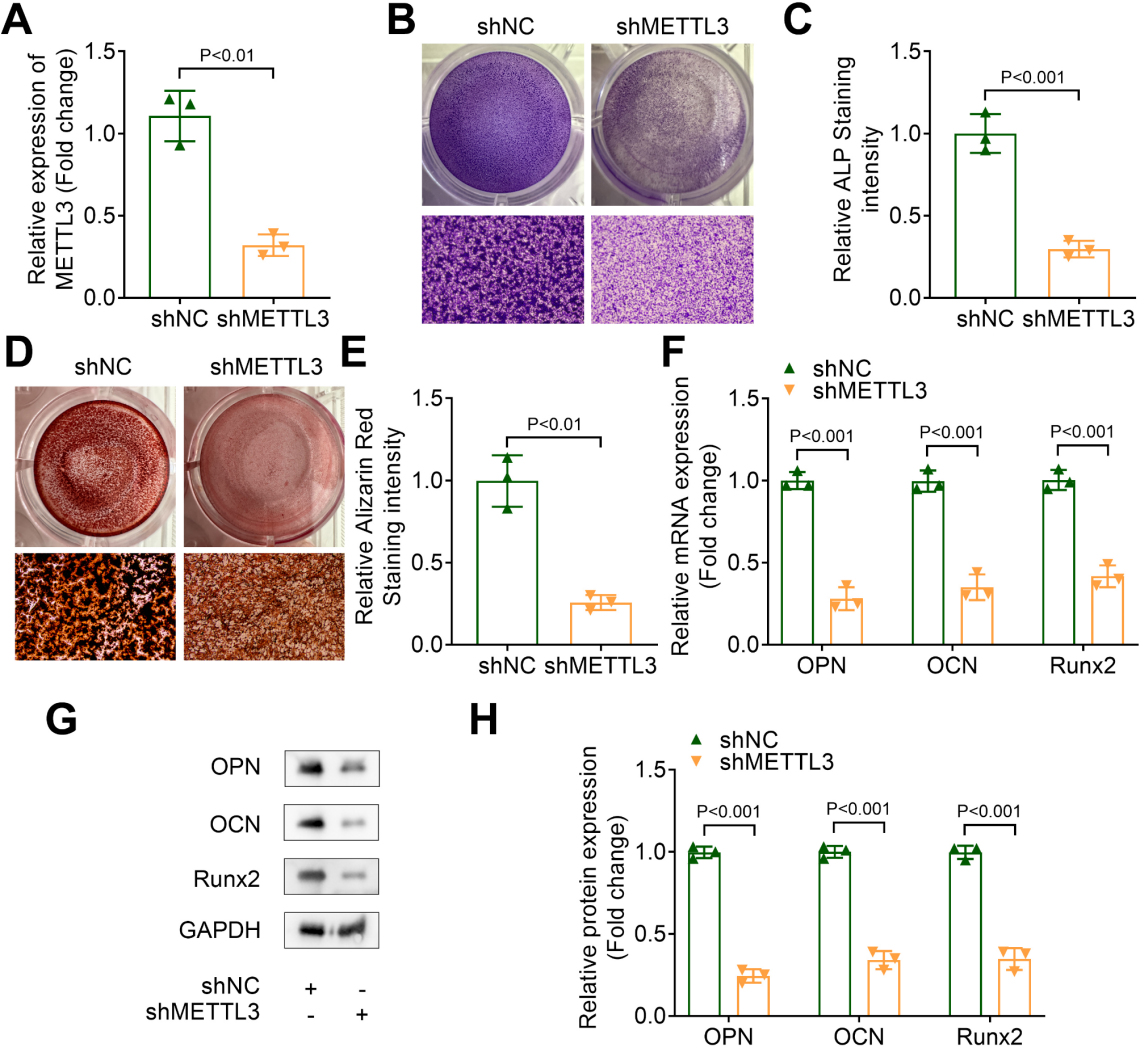
METTL3 knockdown inhibited osteogenic differentiation in OLF cells. (**A**) The expression of METTL3 was elevated by qPCR. (**B** and **C**) ALP staining was performed to assess the ALP staining intensity of OLF cells after transfection. (**D** and **E**) The calcium deposition in OLF cells after transfection was detected by ARS staining. (**F**) The mRNA expression of OPN, OCN and Runx2 was measured by qPCR. (**G** and **H**) Western blot assay was applied to evaluate the protein levels of OPN, OCN and Runx2

To investigate the function of METTL3, OLF cells were transfected with shMETTL3, resulting in a significant reduction in METTL3 expression (Fig. 2A). ALP staining revealed that METTL3 knockdown markedly decreased ALP staining intensity in OLF cells (Fig. 2B, C). Additionally, ARS staining demonstrated that METTL3 knockdown suppressed calcium deposition in OLF cells (Fig. 2D, E). The mRNA and protein levels of osteogenic differentiation markers OPN, OCN, and Runx2 in OLF cells were assessed using qPCR and western blot assays. Our results indicated that METTL3 knockdown downregulated both the mRNA expression and protein levels of OPN, OCN, and Runx2 (Fig. 2F-H). In conclusion, these findings suggested that METTL3 inhibited osteogenic differentiation in OLF cells.

### METTL3 knockdown inhibits m^6^A methylation of BMP2

**Fig. 3:**
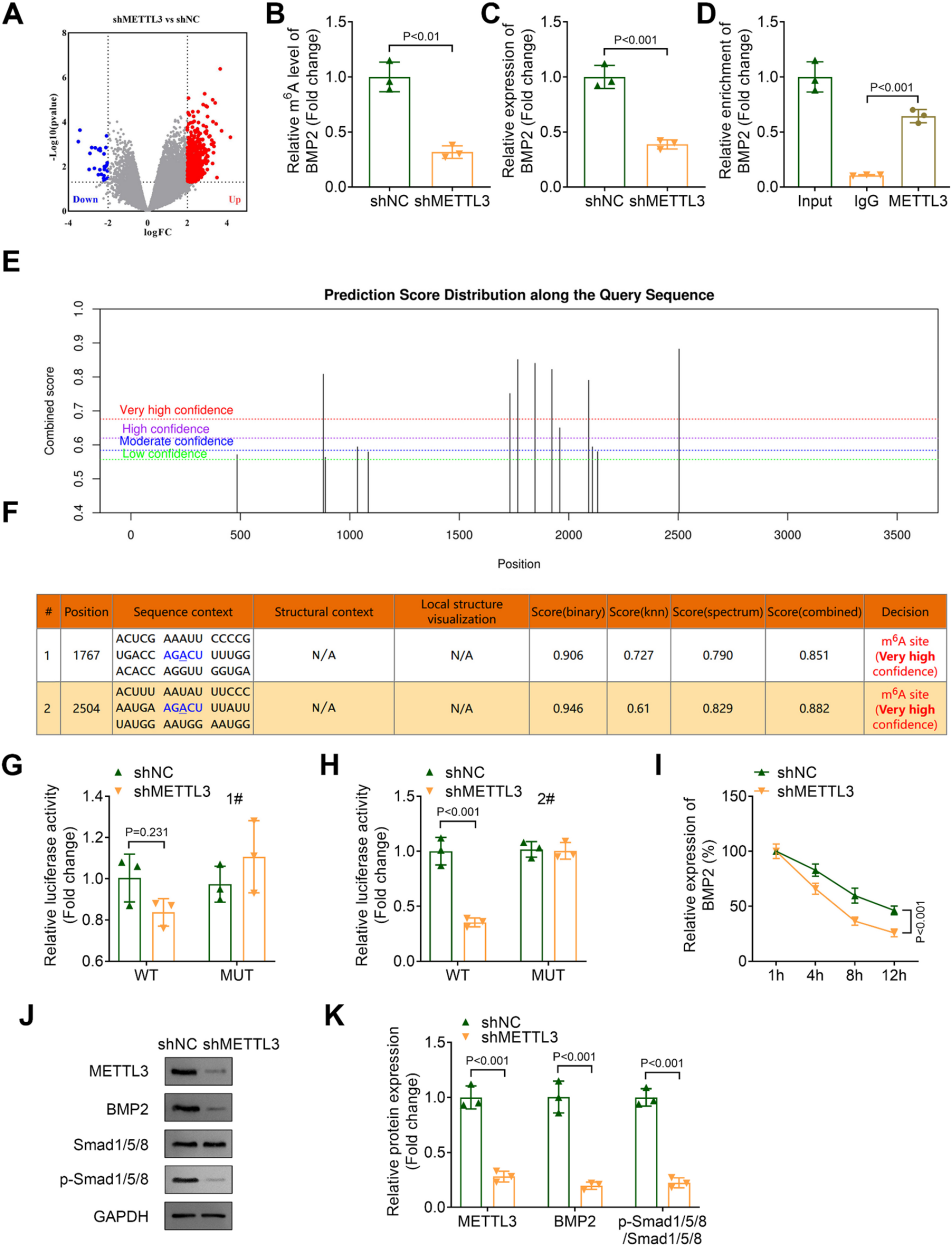
METTL3 knockdown inhibited m^6^A methylation through suppressing the stability of BMP2 (**A**) The DEGs in OLF cells transfected with shNC or shMETTL3 were analyzed by RNA-seq and presented in a volcano plot. (**B**)The m^6^A levels of BMP2 in OLF cells were detected by MeRIP. (**C**) qPCR was performed to measure the expression of BMP2. (**D**) The interaction between METTL3 and BMP2 was determined by RIP. (**E**) The potential m^6^A sites in BMP2 were predicted using the SRAMP database. (**F**) The top 2 sites in BMP2. (**G** and **H**) The luciferase activity of wild type and mutant of site 1 and site 2 in OLF cells co-transfected with shNC or shMETTL3. (**I**) The mRNA expression of BMP2 in OLF cells treated with 5 µg/mL actinomycin D treatment for 1, 4, 8 and 12 h was assessed by qPCR. (**J** and **K**) The protein levels of METTL3, BMP2, Smad/1/5/8, and the phosphorylation levels of Smad/1/5/8 in OLF cells were detected by western blot

Next, we performed RNA-seq to identify DEGs in OLF cells transfected with either shNC or shMETTL3. The results were visualized in a volcano plot (Fig. 3A). A previous study reported that BMP2 regulates the progression of OLF, and our data showed that BMP2 was downregulated in OLF cells upon METTL3 knockdown (Fig. 3A) (Wang et al. 2020). Therefore, we investigated the effect of METTL3 knockdown on m6A methylation of BMP2 in OLF cells. Our results demonstrated that METTL3 knockdown significantly reduced both the m6A modification of BMP2 and its mRNA expression levels (Fig. 3B, C). Then, the interaction between BMP2 and METTL3 was identified using RIP (Fig. 3D). Next, we predicted some potential m^6^A methylation sites of BMP2 (Fig. 3E), and the top two sites are presented in Fig. 3F. Using a dual-luciferase reporter assay, we found that METTL3 knockdown markedly suppressed the luciferase activity associated with WT-site2 (Fig. 3H), while no significant effect was observed on WT-site1 (Fig. 3G). Moreover, compared to the shNC group (BMP2 half-life: 10.62 h), METTL3 knockdown significantly decreased the stability of BMP2 mRNA (half-life: 6.034 h) (Fig. 3I). We then evaluated the impact of METTL3 knockdown on downstream targets of BMP2. Results indicated that METTL3 knockdown not only decreased the protein levels of METTL3 and BMP2, but also reduced the phosphorylation levels of Smad1/5/8 in OLF cells (Fig. 3J and K). Collectively, these findings suggested that METTL3 knockdown inhibited m6A methylation, thereby destabilizing BMP2 mRNA.

### Osteogenic differentiation inhibited by METTL3 knockdown is reversed by BMP2 overexpression

**Fig. 4:**
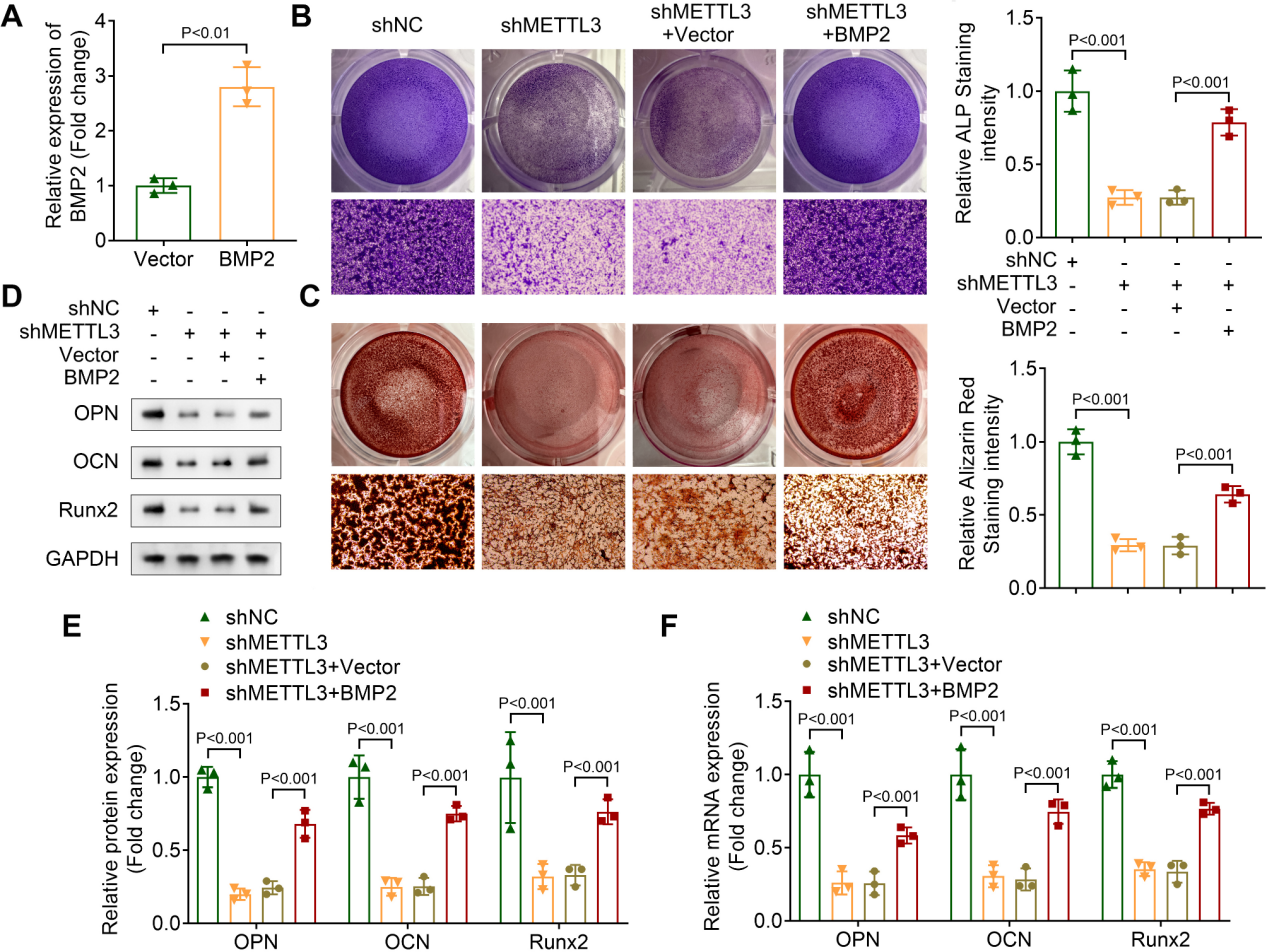
BMP2 overexpression reversed the inhibition of osteogenic differentiation caused by METTL3 knockdown. (**A**) The expression of BMP2 was measured by qPCR. (**B**) ALP staining was performed to assess the ALP staining intensity in OLF cells. (**C**) ARS staining was used to evaluate the calcium deposition in OLF cells. (**D** and **E**) Western blot assay was applied to evaluate the protein levels of OPN, OCN and Runx2. (**F**) The mRNA expression of OPN, OCN and Runx2 was measured by qPCR

To determine the function of BMP2 in osteogenic differentiation, pcDNA3.1-BMP2 was transfected into OLF cells, resulting in increased BMP2 expression (Fig. 4A). ALP staining revealed that the ALP staining intensity suppressed by METTL3 knockdown was partially restored by BMP2 overexpression (Fig. 4B). Additionally, ARS staining demonstrated that BMP2 overexpression increased calcium deposition in OLF cells with METTL3 knockdown (Fig. 4C). These results indicated that BMP2 overexpression partially restored osteogenic differentiation in METTL3-knockdown OLF cells. Furthermore, we assessed the mRNA and protein levels of osteogenic differentiation markers OPN, OCN, and Runx2 in OLF cells. Our findings showed that the reduction in these markers caused by METTL3 knockdown was partially reversed by BMP2 overexpression (Fig. 4D-F). Collectively, these data demonstrated that the inhibition of osteogenic differentiation induced by METTL3 knockdown can be reversed by BMP2 overexpression.

### IGF2BP1 is the m^6^A modification reader of BMP2

**Fig. 5:**
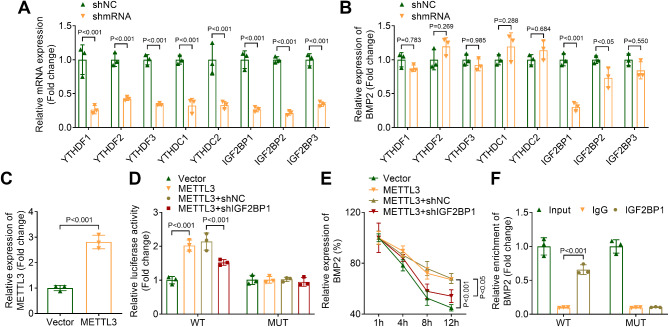
IGF2BP1 was the m^6^A modification reader of BMP2. (**A**) qPCR was performed to measure the expression of m^6^A readers. (**B**) The expression of BMP2 in OLF cells after transfection was evaluated by qPCR. (**C**) The expression of METTL3 in OLF cells transfected with pcDNA3.1 or pcDNA3.1-METTL3. (**D**) Dual luciferase reporter assay was used to measure the luciferase activity of WT-BMP2 and MUT-BMP2 in OLF cells after transfection. (**E**) BMP2 mRNA stability was measured by qPCR after OLF cells were treated with 5 µg/mL actinomycin D for 1, 4, 8 and 12 h. (**F**) RNA pull down was performed to detect the binding between IGF2BP1 and WT or MUT-BMP2 transcript

To identify the m^6^A modification readers of BMP2, OLF cells were transfected with shRNAs targeting YTHDF1, YTHDF2, YTHDF3, YTHDC1, YTHDC2, IGF2BP1, IGF2BP2, and IGF2BP3. The expression levels of these genes were successfully decreased (Fig. 5A). Subsequently, we measured the expression of BMP2 following these transfections. Our results indicated that only the knockdown of IGF2BP1 significantly inhibited BMP2 expression (Fig. 5B). To further investigate the role of METTL3 in this process, we overexpressed METTL3 by transfecting OLF cells with pcDNA3.1-METTL3, resulting in elevated METTL3 expression (Fig. 5C). Dual-luciferase reporter assay suggested that METTL3 overexpression increased the luciferase activity associated with the WT-BMP2 transcript, an effect that was partially reversed by IGF2BP1 knockdown (Fig. 5D). Additionally, compared to the vector control group (BMP2 half-life: 9.544 h, METTL3 overexpression significantly enhanced the stability of BMP2 mRNA (half-life: 19.51 h), an effect that was diminished by IGF2BP1 knockdown (half-life: 12.06 h) (Fig. 5E). Furthermore, RNA pull-down assays confirmed that IGF2BP1 specifically bound to the WT-BMP2 transcript but not to the MUT-BMP2 transcript (Fig. 5F). In conclusion, our findings demonstrated that IGF2BP1 acted as the m6A methylation reader for BMP2.

### Histone lactylation is enhanced in patients with OLF and it promotes METTL3 transcription

**Fig. 6:**
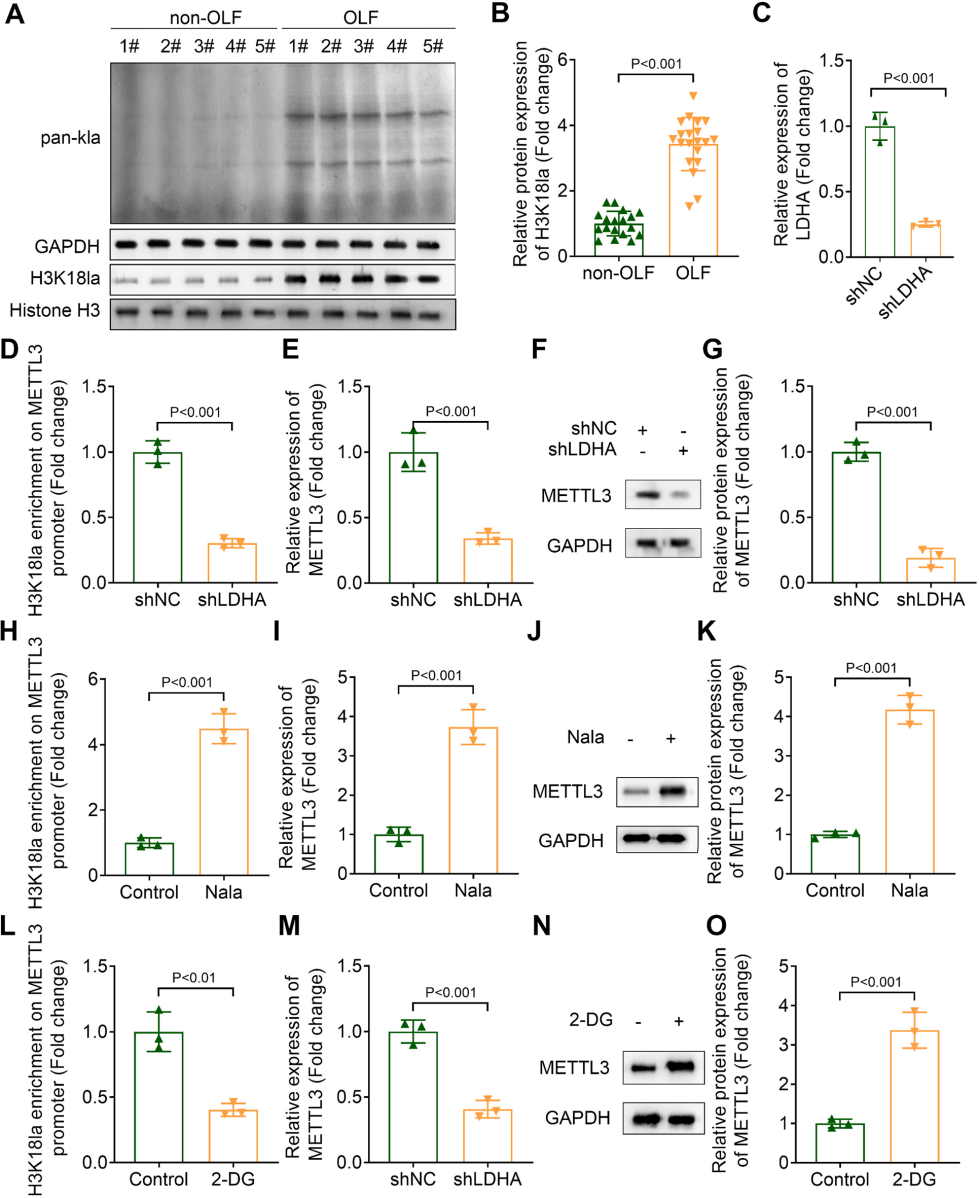
Histone lactylation is enhanced in patients with OLF and it promotes METTL3 transcription. (**A** and **B**) The histone lactylation and pan-kla levels in clinical samples were assessed using western blot assay. (**C**) The expression of LDHA was measured by qPCR. (**D**) The histone lactylation on METTL3 promoter was evaluated by ChIP-qPCR. (**E**) qPCR was performed to measure the expression of METTL3. (**F** and **G**) The protein levels of METTL3 were detected by western blot. (**H**) The histone lactylation on METTL3 promoter in OLF cells with Nala treatment was evaluated by ChIP-qPCR. (**I**) qPCR was performed to measure the expression of METTL3. (**J** and **K**) The protein levels of METTL3 were detected by western blot. (**L**) The histone lactylation on METTL3 promoter in OLF cells with 2-DG treatment was evaluated by ChIP-qPCR. (**M**) qPCR was performed to measure the expression of METTL3. (**N** and **O**) The protein levels of METTL3 were detected by western blot

A previous study identified that histone lactylation mediates osteogenic differentiation (Wang et al. 2023). To investigate this hypothesis, we first examined the protein levels of H3K18la in clinical samples. Compared to patients without OLF, the protein levels of H3K18la and pan-kla were significantly elevated in patients with OLF (Fig. 6A and B), indicating an increased level of histone lactylation in these patients. To explore whether METTL3 is regulated by histone lactylation, we transfected OLF cells with shLDHA, which resulted in a significant reduction in LDHA expression (Fig. 6C). Subsequently, we found that LDHA knockdown inhibited histone lactylation at the METTL3 promoter (Fig. 6D). Furthermore, LDHA knockdown led to a decrease in both the mRNA expression and protein levels of METTL3 (Fig. 6E-G). These results suggested that endogenous inhibition of lactate levels suppresses METTL3 transcription by inhibiting histone lactylation. Next, we increased intracellular lactate levels using Nala. Our data demonstrated that Nala treatment significantly enhanced histone lactylation at the METTL3 promoter, as well as the mRNA and protein expression of METTL3 (Fig. 6H-K). Additionally, we treated OLF cells with 2-DG. The results showed that 2-DG treatment decreased histone lactylation at the METTL3 promoter and reduced both the mRNA expression and protein levels of METTL3 (Fig. 6L-O). In conclusion, our findings suggested that histone lactylation is enhanced in patients with OLF and promoted METTL3 transcription in OLF cells.

### Inhibition of histone lactylation inhibits osteogenic differentiation in OLF cells by downregulating METTL3 expression

**Fig. 7:**
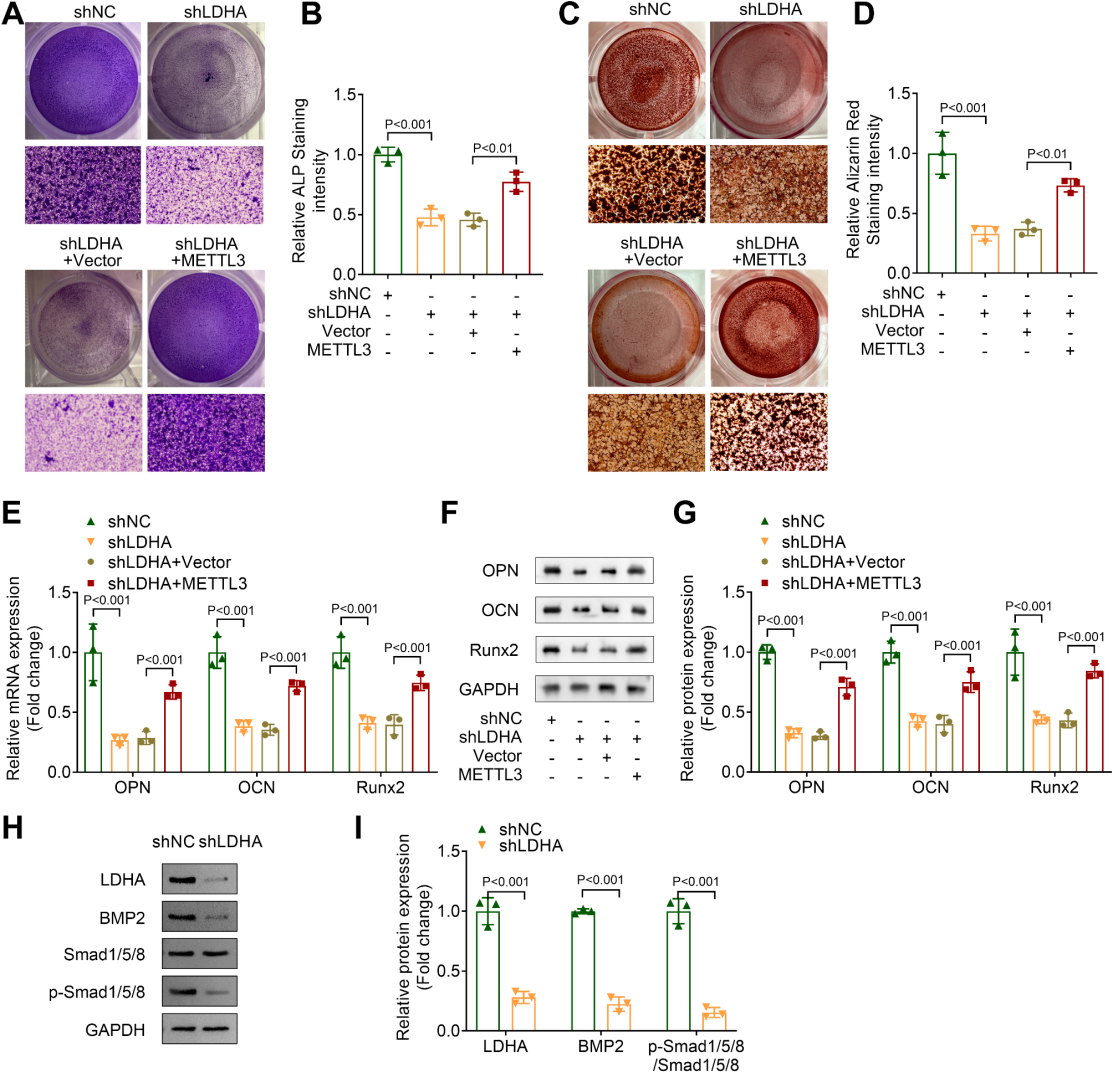
Inhibition of histone lactylation inhibited osteogenic differentiation in OLF cells by downregulating METTL3 expression. (**A** and **B**) ALP staining was conducted to assess the ALP staining intensity. (**C** and **D**) The calcium deposition in OLF cells was evaluated by ARS staining. (**E**) The mRNA expression of OPN, OCN and Runx2 in OLF cells was measured by qPCR. (**F** and **G**) Western blot was performed to measure the protein levels of OPN, OCN and Runx2 in OLF cells. (**H** and **I**) The protein levels of LDHA and BMP2 and the phosphorylation level of Smad/1/5/8 were detected by western blot

Finally, we investigated the effect of histone lactylation on osteogenic differentiation through rescue experiments. ALP and ARS staining results indicated that inhibition of histone lactylation via LDHA knockdown decreased both ALP staining intensity and calcium deposition in OLF cells. However, this inhibitory effect was reversed by METTL3 overexpression (Fig. 7A-D). Furthermore, LDHA knockdown markedly reduced the mRNA expression and protein levels of osteogenic differentiation markers OPN, OCN, and Runx2. This reduction was partially restored by METTL3 overexpression (Fig. 7E-G). Additionally, LDHA knockdown also reduced the protein levels of BMP2 and the phosphorylation level of Smad1/5/8 (Fig. 7H and I). These findings suggest that inhibition of histone lactylation suppresses osteogenic differentiation in OLF cells, at least in part, by downregulating METTL3 expression.

## Discussion

OLF mainly occurs in the thoracic vertebra, and the pathogenesis of OLF is not fully clear. In recent years, studies about the mechanism of OLF progression have been increasing. For example, (Lin et al. 2023) revealed that interleukin-17 A promotes proliferation and osteogenic differentiation of human ligamentum flavum cells through the regulation of β-Catenin signaling. (Yang et al. 2024) reported that M1 macrophage-derived oncostatin M induces osteogenic differentiation of ligamentum flavum cells through the JAK2/STAT3 pathway. These results suggested that inhibition of osteogenic differentiation may attenuate the progression of OLF.

RNA m^6^A methylation has been proven to play an important role in osteogenic differentiation. For example, WTAP-mediated m^6^A modification promotes bone marrow mesenchymal stem cells (BMSCs) osteogenic differentiation (You et al. 2023). METTL3 is a functional protein of m^6^A methylation modification, which shows potential to promote osteogenic differentiation. One recent study demonstrated that METTL3 overexpression partially restores the osteogenic differentiation ability in osteoporotic rats (Wu et al. 2022). Moreover, (Li et al. 2023) revealed that METTL3-mediated LncRNA MIR99AHG methylation enhances the osteogenic differentiation of BMSCs.

On the mechanism of m^6^A modification mediating OLF progression, Wang et al.(Wang et al. 2020) demonstrated that BMP2 modified by the m^6^A demethylase ALKBH5 promotes the ossification of ligamentum flavum. However, whether METTL3 is involved in the development of OLF remains unclear. In this study, we revealed that the METTL3 expression and m^6^A level were increased in patients with OLF. Moreover, METTL3 knockdown inhibited osteogenic differentiation and the expression of osteogenic markers Runx2, OPN and OCN in OLF cells, suggesting that METTL3 may promoted the osteogenic differentiation in OLF.

BMP2 plays a central role in the development and transformation of bone and cartilage, which is also involved in the osteoblastic differentiation of ligamentum flavum cells. Previous studies have shown that the combination of BMP2 and Runx2 promotes the conversion of human ligamentum flavum cells into osteoblastic cells (Kim et al. 2011). Moreover, the expression of BMP2 has been demonstrated to increase in patients with thoracic OLF and thoracic OLF cells (Huang et al. 2022). As previously mentioned, BMP2 modified by ALKBH5 promotes ossification of the ligamentum flavum; however, whether other methyltransferases-modified BMP2 mediates the development of OLF has not been reported yet. In the current study, we found that BMP2 m^6^A level, mRNA expression and stability were decreased by METTL3 knockdown. In addition, the osteogenic differentiation inhibited by METTL3 knockdown was reversed by BMP2 overexpression. These results indicated that the m^6^A modification of BMP2 was mediated by METTL3, and BMP2 overexpression promoted osteogenic differentiation in OLF cells.

In addition, IGF2BP1 was identified as the reader of BMP2 m^6^A methylation in this study. The involvement of m^6^A in the regulation of gene expression depends on the specific recognition of m^6^A-modified RNA by reader proteins (Sun et al. 2024). IGF2BP1 is a member of the m^6^A readers, which is involved in the development of osteogenic differentiation (Huang and Wang 2022). Moreover, IGF2BP1 can enhance the stability of RNA (Huang et al. 2018). Our study demonstrated that IGF2BP1 knockdown partially reversed the luciferase activity of WT-BMP2 and the BMP2 mRNA stability increased by METTL3 overexpression, indicating IGF2BP1 recognized BMP2 m^6^A modification during the regulation of osteogenic differentiation.

Histone lactylation is a new PTM discovered in recent years, and it mediates the development of various diseases. (Wang et al. 2024) investigated that H3K18 lactylation promotes the progression of arsenite-related idiopathic pulmonary fibrosis. (Li et al. 2024) revealed that histone lactylation inhibits RARγ expression in macrophages to promote colorectal tumorigenesis. Some studies revealed the relationship between histone lactylation and osteogenic differentiation. For instance, (Wu et al. 2023) demonstrated that endothelial cell-derived lactate triggers BMSC histone lactylation to attenuate osteoporosis. (Nian et al. 2022) proved that LDHA promotes osteoblast differentiation through histone lactylation. However, whether histone lactylation is involved in the progression of OLF remains uncertain. In this study, we demonstrated that the histone lactylation was increased in patients with OLF. Moreover, LDHA knockdown suppressed osteogenic differentiation in OLF cells, which was partially restored by METTL3 overexpression. These results indicated that inhibition of histone lactylation restrained osteogenic differentiation in OLF cells through suppressing the expression of METTL3.

While our study demonstrates that METTL3 regulates osteogenic differentiation in OLF via m6A methylation of BMP2, several limitations should be acknowledged. We acknowledge that BMP2 overexpression only partially rescues the phenotype caused by METTL3 deficiency, indicating that other pathways or molecular mechanisms might be involved. For instance, METTL3 may co-regulate osteogenesis by targeting m6A modifications of other osteogenic genes or interacting with non-coding RNAs that modulate bone formation. Furthermore, histone lactylation might influence OLF progression through METTL3-independent pathways, such as regulating additional epigenetic modifiers or metabolic regulators. Future investigations employing multi-omics approaches are warranted to systematically identify METTL3 targets and elucidate their crosstalk with BMP2 signaling. These efforts will refine our understanding of the complex regulatory network underlying OLF pathogenesis.

In conclusion, we revealed that histone lactylation-mediated METTL3 promoted the progression of OLF by mediating m^6^A methylation of BMP2, which may provide a novel therapeutic target for OLF.

## Data Availability

The datasets used and/or analysed during the current study are available from the corresponding author on reasonable request.
